# Seroma control in axillary lymphadenectomy with Glubran 2® without drain. Multicenter, prospective, randomized, clinical trial. GALA-ND study (Glubran, Axillary Lymphadenectomy, Ambulatory, No Drain)

**DOI:** 10.1186/s13063-023-07840-w

**Published:** 2024-02-22

**Authors:** Sandra López Gordo, Neus Ruiz-Edo, Maria Teresa Fernández-Planas, Sara Viscaya-Martín, Cristina Serra-Serra, Breast Cancer Research Group, Carmen Buqueras, Mireia Recaj, Raul Guerrero-López, Lidia Blay-Aulina, Oscar Aparicio-Rodriguez, Laura Cusiné, Xavier Mira, Montserrat Clos, Immaculada Alonso, Jairo Cortés Prados, Sofía Espinoza Villalobos, Elena Vallejo-Barnosell, A. S. Espinoza-Villalobos, Marta Jimenez, Aurora Carrasquer-Puyal, Priscila Giselle Holub, Maria José Cases Baldo, E. Garcia de Castro R, Inmaculada Herrador Garcia, Emanuela Esposito, E. Garcia de Castro Rubio, Marta Lourdes Gonzalez Duaigues

**Affiliations:** 1 General Surgeon, Breast Cancer Unit of Maresme Health Consortium (Mataró Hospital), Carr. de Cirera, 230, 08304 Mataró, Barcelona, Spain; 2 Associated professor at the Autonomous University of Barcelona (UAB), Bellaterra 08193, Barcelona, Spain; 3 Present Address: Radiologist, Breast Cancer Unit of Maresme Health Consortium (Mataró Hospital), Carr. de Cirera, 230, 08304 Mataró, España; 4 Gynecologist, Breast Cancer Unit of Maresme Health Consortium (Mataró Hospital), Carr. de Cirera, 230, 08304 Mataró, España; 5 Hospital Penna, Buenos Aires, Argentina; 6 Hospital Virgen del Rocio, Sevilla, Spain; 7 Hospital Arnau de Vilanova, Lleida, Spain

**Keywords:** Seroma, Sealant, Axillary lymphadenectomy, Drain, Breast cancer

## Abstract

**Background:**

Seroma after breast cancer surgery is a frequent entity; therefore, different products have been described in literature with the aim to reduce it. The most studied ones have been the sealants products, being tested with aspirative drains. Symptomatic seroma represents the 19% after axillary lymphadenectomy without drains. The aim of this study is to analyze the effect of a sealant in the seroma control after axillary lymphadenectomy without drains and identify the risk factors related to symptomatic seroma.

**Methods:**

This is a prospective, multicenter, international, and randomized clinical trial. Patients undergoing conservative surgery and axillary lymphadenectomy for breast cancer will be randomized to control group (lymphadenectomy without sealant) or interventional group (lymphadenectomy with sealant Glubran 2®). In any of the study groups, drains are placed. Patients who received neoadjuvant treatment are included. Measurements of the study outcomes will take place at baseline; at 7, 14, and 30 days post-surgery; and at 6–12 months. The primary outcome is symptomatic seroma. Secondary outcomes are seroma volume, morbidity, quality of life, and lymphedema.

**Discussion:**

Several studies compare the use of sealant products in axillary lymphadenectomy but generally with drains. We would like to demonstrate that patients who underwent axillary lymphadenectomy could benefit from an axillary sealant without drains and reduce axillary discomfort while maintaining a good quality of life. Assessing the relationship between axillary volume, symptoms, and related risk factors can be of great help in the control of seroma in patients who received breast cancer surgery.

**Trial registration:**

ClinicalTrials.gov, NCT05280353. Registration date 02 August 2022.

## Administrative information

**Table:** 

Title {1}	Seroma control in axillary lymphadenectomy with Glubran 2® without drain. Multicenter, prospective, randomized, clinical trial. GALA-ND study (Glubran, Axillary Lymphadenectomy, Ambulatory, No Drain)
Trial registration {2a and 2b}	ClinicalTrials.gov, NCT05280353 The Universal Trial Number (UTN) U1111-1285–0079
Protocol version {3}	Date 16/03/2022 version 3
Funding {4}	Glubran 2® product was financed by Cardiolink Group This research benefits from a research grant from the Spanish Association of Surgeons (Asociación Española de Cirujanos, AEC)
Author details {5a}	Breast Cancer Unit of Maresme Health Consortium (Mataró Hospital)
Name and contact information for the trial sponsor {5b}	Cardiolink Group S.L. Juan Ruiz, Director division. Telf. + 34 669,097,937 Web: www.cardiolinkgroup.com AEC: Calle O´Donnell, 16. 1° izq. 28,009 – Madrid. Telf: (+ 34) 91 319 04 00
Role of sponsor {5c}	Cardiolink Group S.L: Payment for the use of the Glubran 2® product used in all hospitals participating in the study. Cardiolink Group S. L also contributed to the design of the study, the collection, analysis and interpretation of data, and to writing of the manuscript AEC: Payment of the fees for publication of the protocol study

## Introduction

### Background and rationale {6a}

Breast cancer (BC) represents a major health problem due to its high incidence, being the most frequent cancer in women and the most diagnosed cancer worldwide.

According to data from the Spanish Society of Medical Oncology [1], a total of 34,750 new cases of BC were diagnosed in Spain in 2022 with a 5-year prevalence of 144,233 in 2020 [1]. The estimated number of new BC cases for 2022 is 34,750 [1].

Axillary involvement has classically been considered the most important prognostic factor in BC. For decades, axillary lymphadenectomy (AL) has been performed for the purpose of staging, control, and improved survival in patients with BC. The clinical practice of surgical management of the axilla underwent a major change following the study published by Giuliano et al. [2], with a decrease in the number of ALs performed worldwide. Today, AL is still the treatment of choice in patients with node involvement (pN +), both those undergoing primary surgery and after neoadjuvant treatment, being this last scenario controversial to date.

However, the morbidity of AL is not negligible. Different series report the following prevalence of complications associated with the surgical technique: chronic lymphedema in the upper extremity (20–30%), seroma (50–60%), wound infection (5–15%), frozen shoulder syndrome (up to 10%), neuropathic pain due to intercostobrachial nerve injury (5–20%), and other less frequent complications such as hematoma, scapular winging (long thoracic nerve injury), or latissimus dorsi muscle atrophy (latissimus dorsi bundle injury).

The most common complication after AL is seroma (15–90% depending on series) [3]. Seroma is defined as the presence of serous fluid in the areas of surgical resection or dissection. Seroma is mainly seen after mastectomy and AL. Although the basis of seroma formation is not fully understood, factors such as surgical technique, extent of dissection, and sealing devices used may influence its control [4].

The usual clinical practice in these procedures includes the placement of an axillary aspirative drain, which is usually removed when the amount of drainage collected is low enough. However, this clinical practice is questioned by some authors [5]. Given its high incidence, several groups have made efforts to achieve a reduction and better control of seroma [3], noting that most of them use suction drains after surgery. According to the conclusions published by Cochrane in 2013, there is limited evidence that the insertion of a drain after axillary lymphadenectomy reduces the likelihood of developing seroma and/or reduces the number of aspirations needed to control it [5]. Given the current evidence regarding the use of drains after lymphadenectomy, in our study, we decided not to use drains after regular axillary lymphadenectomy.

Efforts to control and reduce the seroma volume after AL and therefore help our patients improve their recovery and have a better QoL have been made but without any significant results that lead us to change clinical practice yet. Some teams suggest the use of sealants and/or biological glues [6]; however, to date, there are still no relevant results to allow clinical practice to change [7]. Studies assessing the use of such sealants include patients with and without drains. Currently in breast cancer, percentages close to 20–25% of symptomatic seroma requiring drains are described [8, 9].

There is a large amount of conflicting scientific evidence regarding current management of seroma after AL. However, there is a lack of evidence stablishing a relationship between the volume of the axillary seroma and its symptoms. Moreover, the effect of tissue sealants in seroma control without using suction drains has not been sufficiently investigated either. For this reason, the present study is a randomized multicenter international clinical trial that aims to analyze the role of a tissue sealant in seroma production and in the control of pain-related-to-seroma in patients with BC undergoing LA without a drain placement.

Glubran 2® is a synthetic surgical glue with a cyanoacrylic base modified by the addition of a monomer synthesized by the manufacturer, approved for its use in surgery for its hemostatic, sealing, and adhesive properties that facilitate strong tissue adhesion. Its active ingredient is N-butyl-2-cyanoacrylate + methacrylosulpholane.

Some authors consider and demonstrate that after reducing the axillary space in AL, either by means of sealants or by a surgical approximation of tissues, a reduction in seroma is achieved. However, there is even evidence of a reduction in the percentage of seroma in patients in whom Glubran 2® is applied after breast cancer surgery [10].

We have designed the current study with the product Glubran 2® to demonstrate that, through its sealing and adhesive properties at axillary level, could reduce the axillary seroma rate.

## Objectives {7}

### Main objectives

1. To evaluate the efficacy of Glubran 2® in reducing the percentage of symptomatic seroma after AL without drain
2. To assess the safety and tolerability of Glubran 2® in breast cancer surgery after AL without drain

### Secondary objectives

1. To assess whether there are differences in axillary seroma volume and its relationship to symptomatic seroma between groups
2. To assess whether there are differences in the number of punctures required, as well as the volume aspirated between groups
3. To assess the existence of risk factors related to symptomatic seroma
4. To compare morbidity between study groups in the short and long term
5. To assess whether there are differences in seroma volume, symptomatic seroma, and final nodal anatomy between groups
6. To assess quality of life (QoL) after the intervention as measured by the EQ-5D-5L questionnaire (Spanish version)

### Main hypothesis

The use of Glubran 2® after AL in patients with BC can safely reduce the symptomatic seroma percentage.

### Justification

The administration of an axillary sealant after AL (without the use of drain) could reduce the number of symptomatic seroma and therefore require fewer axillary punctures/evacuations, thus improving the quality of life of patients.

### Trial design {8}

**Fig. 1:**
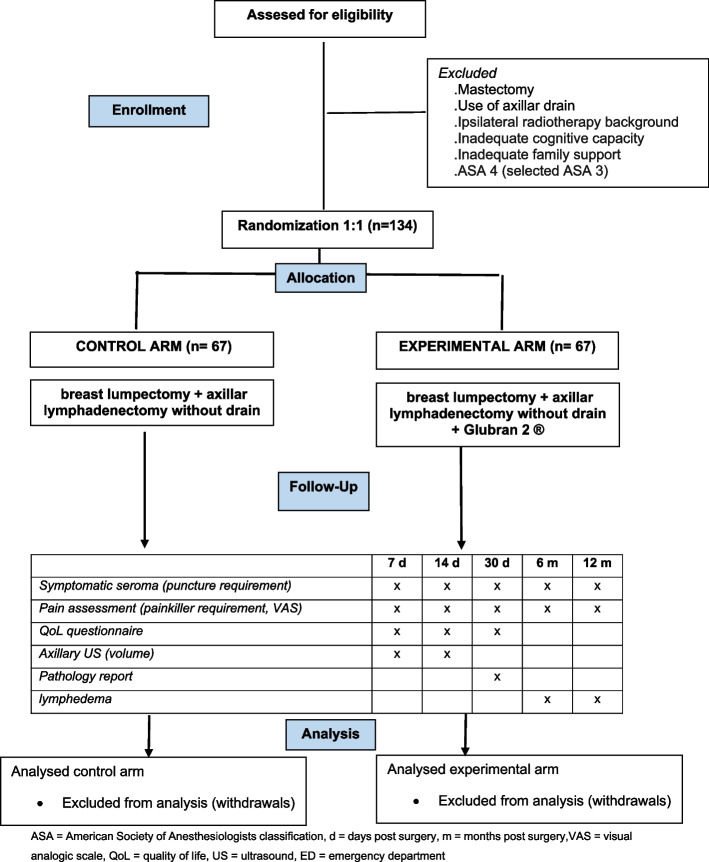
CONSORT Flow diagram

This is a multicenter, international, prospective, double-arm randomized clinical trial with a superiority design. Baseline data will be measured prior to randomization. Follow-up data will be collected at 7, 14, and 30 days after randomization. Long follow-up data will be collected at 6 and 12 months. A CONSORT diagram is presented in Fig. 1.

## Methods: participants, interventions, and outcomes

### Study setting {9} CHUMI (Canarias)

Data will be collected from patients treated mainly in Spanish hospitals but also in Argentina and Italy, giving this study an international approach. Participant hospitals are as follows: Maresme Health Consortium (Mataró Hospital), Moisès Broggi Sant Joan Despí Hospital, University Hospital Mútua de Terrassa, Corporació de Salut del Maresme i la Selva, University Hospital Vic, Can Ruti University Hospital, Corporación Sanitaria Parc Tauli, Hospital University d’Igualada, Granollers General Hospital, Santa Creu i Sant Pau University Hospital, Joan XXIII University Hospital, Consorci Sanitari of Terrassa Hospital, Parc Salut Mar University Hospital, Barbastro Hospital, Vega Baja Hospital, Severo Ochoa Hospital (Madrid), Istituto Nazionale Tumori, IRCCS, Fondazione Pascale, Napoli,. Virgen del Rocio Hospital, Arnau de Vilanova Hospital and Penna Hospital.

### Eligibility criteria {10}

Inclusion criteria

- Over 18 years old
- Conservative surgery for BC with associated AL

Exclusion criteria

- Need for mastectomy
- History of ipsilateral axillary radiotherapy
- ASA 4 patients (selected ASA 3 patients)
- Lack of adequate cognitive capacity and/or failure to sign the informed consent form

In all the cases, surgeons (general surgeon or a gynecologist) will perform the intervention during breast cancer surgery.

### Who will take informed consent? {26a}

During the standard preoperative clinical assessment, all BC patients who are candidates for surgery meeting the inclusion criteria will be informed of the possibility to participate in the present study by the surgeon. The patient will give their authorization by signing an informed consent document during the base-line visit.

### Additional consent provisions for collection and use of participant data and biological specimens {26b}

Patients enrolling this study gave informed consent to be followed-up during the study period and to allow the use of their medical records according to the trial’s objectives, for academic purpose. This trial does not include biological specimens.

## Interventions

### Explanation for the choice of comparators {6b}

Symptomatic seroma is the comparator chosen to prove if a sealant has an effect after AL in patients without drain. All patients are treated following the current breast cancer guidelines; therefore, the criteria for performing AL are uniform among participating hospitals. Pain is a usual symptom after axillary surgery; however, we wanted to evaluate if there is the use of a sealant agent has any role in pain control in this patients. We chose the QoL EQ-5D-5L questionnaire because it also allows to check the upper extremity mobility.

### Intervention description {11a}

The AL will be performed in all cases with a sealing device such as LigaSure®, Harmonic Focus®, or similar. The incision may be chosen by the surgeon at each of the participating centers. AL will be performed according to standard technique, including Berg levels I and II and level III if necessary.

After AL is performed, Glubran 2® will be placed in the axillary hollow in the study group. The product will be applied homogeneously in all participating centers. For this purpose, training lessons have been held in each hospital, with both visual material and the presence of a product expert on the field. After washing the armpit with physiological saline, assuring it is completely dry and caring for an exhaustive control of hemostasis, the product should be applied using its specific applicator (yellow). It has to be distributed over the entire lymphadenectomy area including the following: the area above the axillary vein, the area below the axillary vein, around the latissimus dorsi bundle, the lateral costal wall including the vascular-nerve bundle, the serratus muscle, and the subcutaneous cellular tissue. The closure of the subcutaneous cellular tissue and the skin will be led to the standard in each participating center, although the most common technique is the closure of the subcutaneous cellular tissue with simple stitches of resorbable multifilament suture and the closure of the skin with a continuous intradermal suture of resorbable monofilament thread.

### Hospitalization regime and analgesic regimen

Patients will be operated on an outpatient or conventional inpatient basis, according to the criteria of each hospital participating in the study.

The recommended analgesia at discharge will consist of paracetamol 1g/8 h alternating with metamizole 575 mg/8 h for the first 7 postoperative days in all patients. The need to extend this regimen will be assessed at the different clinical follow-up visits. In case of allergy or intolerance, the regimen will be adapted with two alternating analgesics.

The performance of a neurological block prior to surgery will be recorded.

### Criteria for discontinuing or modifying allocated interventions {11b}

In accordance with the Declaration of Helsinki, participants can withdraw from the trial at any time and for any reason.

The withdrawal criteria is as follows: patients will be excluded from the study in any of the following scenarios: (a) the patient expresses their willingness to leave the study, (b) the patient is not able to accomplish the established clinical controls, (c) continuation of the study procedures may be detrimental to the patient’s health or well-being, (d) there is a concurrent illness that prevents compliance with patient monitoring and assessment procedures.

### Strategies to improve adherence to interventions {11c}

A breast cancer nurse in each center will be the responsible to check that the clinical visits and ultrasound sessions are met and in the adequate timing. They will also be the contact person for patients in case of doubts about their condition or any questions related to the study.

### Relevant concomitant care permitted or prohibited during the trial {11d}

All aspects of clinical care will be permitted as long as they are registered in the medical records.

### Provisions for post-trial care {30}

If any side effect occurs due to the use of Glubran 2® or any other action concerting the study, it will be covered by the hospital’s insurance.

## Outcomes {12}

### Primary outcome

Percentage of patients with symptomatic seroma, defined as a seroma requiring its puncture-aspiration.

Seroma puncture-aspiration is indicated in the following scenarios during follow-up:

- Tension seroma causing uncontrollable pain (VAS = 5) even though taking the recommended oral analgesia (paracetamol 1 g/8 h alternating with metamizole 575 mg/8 h)
- Seroma volume not allowing a full adduction of the upper limb and causing discomfort as a result

### Secondary endpoints

1. Number of seroma aspiration-punctures required
2. Pain (measured by visual analogue scale, VAS)
3. Axillary seroma volume (measured by axillary ultrasound in cm.^3^)
4. Number of post-operative emergency room visits
5. Need for hospital readmission
6. Presence of lymphoedema in the arm
7. QoL after the intervention (measured by the EQ-5D-5L questionnaire, Spanish version)

### Main safety measures

The main safety measures are as follows: adverse events and post-surgical complications such as suture failure and surgical wound infection.

### Other study variables

1. Surgical wound infection: defined as the presence of inflammatory signs—heat, redness, increased temperature and/or pain—requiring antibiotic treatment with or without surgical or percutaneous drainage
2. Surgical regimen: major outpatient surgery [11] or short-stay hospitalization (first 24–48 h post-operatively)
3. Anesthetic technique performed. Use of neuromuscular blockade prior to surgery
4. Definitive pathological anatomy (number of axillary lymph nodes)

### Participant timeline {13}

After surgery, all patients in the study will follow the same clinical assessments, which will be at days 7, 14, and 30 post-surgery. Patients will also be evaluated in the long term, at 6 and 12 months after surgery (Table 1). Clinical visits outside those that are established will be made at the discretion of the surgeon at each participating center.

**Table 1:**
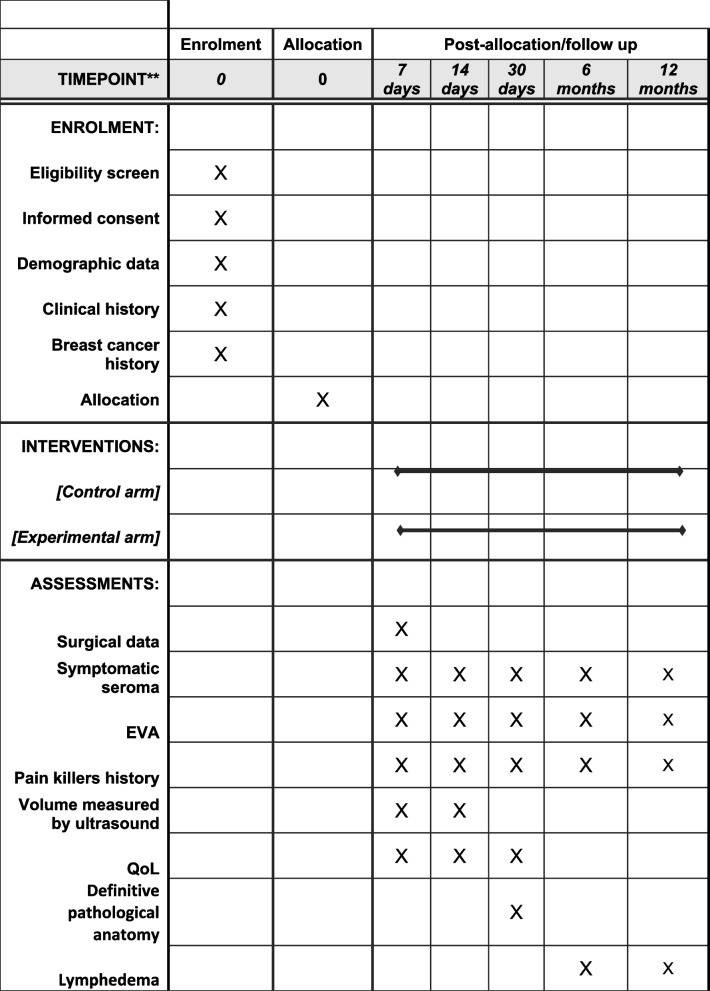
Schedule of enrolment, interventions, and assessments

During clinical assessments, data on the level of pain (VAS), adherence to prescribed analgesic medication, presence of symptomatic seroma, need for puncture-evacuation of the seroma, and QoL will be collected.

### Sample size {14}

Accepting an alpha risk of 0.05 and a beta risk of 0.2 in a bilateral contrast, 67 subjects in each study arm are required to detect a statistically significant difference in the percentage of patients with symptomatic seroma (which is expected to be 25% for the control group and 7% for the study group). A loss to follow-up rate of 10% has been estimated.

For the calculation of “*n*” and due to the large extent of seroma described in the literature, reference values of 25% described in the study by Jan et al. [9] have been taken.

### Recruitment {15}

All the hospitals participating in this trial have a breast cancer unit, all of them treating more than 100 BC patients a year. Hospitals without a breast cancer unit are not included.

The recruitment of patients must end by December 2024. For this reason, the hospitals included in the trial can be expanded during 2023 with the objective to include the required number of centers in each arm. The minimum number of patients required in each hospital is 5, and the maximum is 10.

The main investigator from each hospital is responsible for identifying potential patients who meet the study inclusion criteria. If there is any patient who required a conservative treatment in the breast and a lymphadenectomy, the main investigator must inform the patient about the trial.

## Assignment of interventions: allocation

### Sequence generation {16a}

The allocation of participants to the different groups has been carried by means of a computer-generated randomization stratified by center.

### Concealment mechanism {16b}

Randomization lists have been prepared using the REDCap (Research Electronic Data Capture) program. At the time of recruitment, each participant is assigned a personal and non-transferable study identification number.

REDCap is a secure and customizable web-based database application used to collect and store research data from case report forms, surveys, or a combination of both.

### Implementation {16c}

The surgeon will enter the affiliation data of the patient within the REDCap program. If the inclusion criteria in the trial are met, the program will randomize the patient. The surgeon will be the only person that knows the group in which the patient is allocated. The main investigator of each hospital will be able to review what arm each patient has been assigned to.

## Assignment of interventions: blinding

### Who will be blinded {17a}

Patients, trial statisticians, and postoperative care nurses will be blinded after assigning the intervention.

### Procedure for unblinding if needed {17b}

In case of withdrawal from the trial, the patient will be able to know in with arm of treatment was allocated.

## Data collection and management

### Plans for assessment and collection of outcomes {18a}

For each patient included in the study, a patient case report form (CRF) will be filled in. This also applies to patients who do not complete the full follow-up foreseen in the trial. Subjects will not be identified by name or initials in the CRF or in any trial document. The only acceptable identification that will appear on the CRF or other documents is the subject’s unique subject identification number. The investigator will retain the contact details of all participants, so that they can be contacted quickly if necessary.

Data will be collected via a study-specific electronic case report form (eCRF) by using REDCap program. This eCRF include the necessary fields to fill in during every visit (7, 14, and 30 days and 6 and 12 months). Final data will be cheeked by an independent data monitoring committee which will verify the veracity of the data and the absence of duplications. The database will only be locked for the final analysis, after all data management and statistical data validation checks have been satisfactorily resolved.

QoL will be measured by EQ-5D-5L questionnaire (Spanish version) [12]. This questionnaire has been validated by EuroQol in 2009. EQ-5D-5L measures mobility, self-care, usual activities, pain/discomfort, and anxiety/depression, with four levels of severity in each dimension: no problem, some problems, serious problems, or disability.

Pain is measured by the visual analogue scale (VAS), ranging from 0 to 10.

The REDCap program makes it easy to fill in the patient data without errors in measurements, since if they are not correct, it does not allow them to be recorded.

Data in the eCRF will be checked by the main investigator before the extraction. Data also will be extracted by the statisticians and double checked to avoid duplication or typographical errors.

### Plans to promote participant retention and complete follow-up {18b}

The nurses of each breast cancer unit will be the ones checking that the patients have the appointments established according to the trial.

### Data management {19}

Data registered in the eCRF will be filled in by the surgeon in charge of each patient. An independent data monitoring committee will, at the end, check all data to make sure there are no duplications or other errors. The main investigator of the trial will also validate data periodically. Data discrepancies will be flagged to the study site, and any data changes will be recorded to maintain a complete audit trail (reason for change, date when the change was made, and who made change).

### Confidentiality {27}

The patient’s clinical data shall be completely dissociated from any information that would allow identification of the patient. In all reports and communications relating to trial subjects, the subject will be identified only by case number. The main investigator of each hospital (surgeon) will be the one to register all data in the eCRF; besides him, no one else will have access to it. All data will be registered in fields in REDCap program with a single password, so that no one apart from main investigator can open it.

This file will be treated strictly according to professional standards of confidentiality and will be archived under appropriate security measures and restricted access, in the terms provided for in Regulation [11] 2016/679 of the European Parliament and of the Council of 27 April 2016 on Data Protection (RGPD) as well as in the Organic Law 03/2018, of 5 December.

### Plans for collection, laboratory evaluation, and storage of biological specimens for genetic or molecular analysis in this trial/future use {33}

This trial does not include biological specimens.

## Statistical methods

### Statistical methods for primary and secondary outcomes {20a}

The statistical analysis plan (SAP) will be based on intention to treat principles in line with Consolidated Standards of Reporting Trials (CONSORT) guidelines.

Descriptive analysis of the study sample is as follows: as quantitative variables, the mean, median, range, and standard deviation will be analyzed. Absolute values and percentages will be used for qualitative variables. The frequency measures used will be prevalence, analysis of the homogeneity, or comparability of study groups. Quantitative variables will be assessed for normal distribution using the Kolmogorov–Smirnov or Shapiro–Wilk test.

Bivariate analysis of quantitative variables will be performed using Student’s *t*-test or Mann Whitney *U* test as appropriate.

For the analysis of qualitative variables, the chi square test or Fisher’s test will be used as appropriate. The OR (odds ratio) is considered as a measure of effect calculated by logistic regression.

In the multivariate survival analysis, the Cox regression method will be used, while for the multivariate analysis of risk factors, the logistic regression method will be used, introducing in the statistical model those variables that show statistical significance in the univariate analysis and/or those that in the bibliography and theoretical framework are prognostic factors.

Clinical lymphoedema-free survival will be analyzed using the Kaplan–Meier method, and survival curves will be compared using the Long-Range test and Cox regression. The safety analysis will be performed per protocol and will use the same statistical tests mentioned above. The level of significance is set at *p* < 0.05. The statistical analysis will be carried out with the Statistical Package for the Social Sciences (SPSS) version 21.

### Interim analyses {21b}

No interim analysis is planned in the current study.

### Methods for additional analyses (e.g., subgroup analyses) {20b}

Analyses of the primary and secondary outcome will be done in relation to subgrouping variables including age, sex, type of tumor, neoadjuvant treatment, and pathological history.

For each subgroup analysis, we will undertake a Cox proportional hazards model assessing each primary outcome incorporating a subgroup interaction term to provide the basis for evaluating subgroup effects. We will consider the possibility that a subgroup effect is present if the interaction term of treatment and subgroup is statistically significant at a *p*-value < 0.05. We will also consider other credibility criteria to judge the reliability of a subgroup effect.

### Methods in analysis to handle protocol non-adherence and any statistical methods to handle missing data {20c}

Patients who are included in the study but who do not meet the inclusion or exclusion criteria will be withdrawn from the analysis. Missing data will be excluded for the primary and secondary outcome analyses. Further analyses based on multiple imputation methods will be considered if appropriate. Analyses will be carried out based on the intention to treat principle.

### Plans to give access to the full protocol, participant level-data and statistical code {31c}

Every main investigator will receive a full protocol copy and could also visualize it in REDCap program. In case that a patient would like to consult it, the surgeon will be the one in charge to provide such information. Data will not be publicly available; only the main investigator of each hospital, statistician, and data monitoring committee could visualize it.

The use of data and statistical code is subject to the main investigator decision after signing a written agreement.

## Oversight and monitoring

### Composition of the coordinating center and trial steering committee {5d}

The main research or project team of the trial is created and formed by three surgeons who work in the coordinating center. The project team has the responsibility to control the hospitals included in the trial (provide information, evolution of the inclusion of patients, evolution of CEIM acceptance, and resolve any doubts), control all the patients included in the main investigator hospital, modify the protocol if required and present the modification/s to the ethic committee, and revise the veracity of the data included in the REDCap program.

The trial steering committee is responsible for monitoring and supervise the progress of the study towards its interim and overall objectives. Contact with the main research team must be easy and affordable. The trial steering committee is formed by the main investigator of the trial, the chief of the research unit, a statistician, and the sponsor Cardiolink Group S.L.

The main investigator of each hospital included in the trial is responsible for the patients included in his organization, for verifying the presence of a right informed consent, and for ensuring a correct recruitment of patients and veracity of the data.

The main investigator of the coordinating center will double check all the information and documents provided by the rest of the centers.

### Composition of the data monitoring committee, its role and reporting structure {21a}

An independent data monitoring committee (iDMC) has been created with independent members (clinical, statistical, and methodological expertise). The iDMC is independent from the sponsors and depends from the coordinating center.

### Adverse event reporting and harms {22}

Throughout the study, every effort should be made to detect and evaluate adverse events or harmful findings. If adverse events occur, the primary concern is for the safety and well-being of the subject. Appropriate medical intervention will be undertaken. This includes all adverse events or complications observed by the investigator or reported by the subject, whether or not caused by the surgery under study. The relationship of the adverse event to the surgery will be assessed by the investigator and documented in the case report form (CRF). The investigator should complete the entire CRF and notify the sponsor for review, including a description of the event, the date of onset, an assessment of its relationship to the surgery, a medical assessment of its severity, the actions taken, whether the study surgery had to be discontinued, and the resolution of the event. Serious adverse events that continue to exist at the end of the study period should be followed up to determine the final outcome. Any adverse event that occurs after the study period and is considered possibly related to the study surgery or study participation should be recorded and reported immediately. A pre-existing condition, i.e., one that was present at the start of the study, should be recorded as an adverse event if its frequency, intensity, or nature worsens during the study period.

The sponsor is responsible for adverse event classification and ongoing safety assessment of the clinical investigation and shall:

- Review the investigator’s assessment of all adverse events and document in writing their severity and relationship to the experimental intervention. In case of disagreement between the sponsor and the principal investigator, the sponsor shall communicate both opinions to the Investigation Ethics Committee (IEC) and to the national competent authorities, if necessary
- Report or ensure that the principal investigator reports, all serious adverse events to the IEC. The investigator should report any serious adverse events to the sponsor and the local IRB as soon as he/she becomes aware of them, by fax/telephone
- Report all serious adverse events to the competent authorities within the required time period, if required by national laws
- Inform all local principal investigators, in writing, of all serious adverse events reported to the sponsor and ensure that they report them to their IECs if required by national law. This information should be sent to all principal investigators within a time period established on the basis of potential risk
- Ensure that any new information on the clinical investigation is communicated to the competent authorities

### Frequency and plans for auditing trial conduct {23}

The project team met every week at the beginning of the project. After the elaboration of the trial and the start of the inclusion of the first patients, meetings will take place every month or more often (ad hoc) if required.

The trial steering committee will initially meet monthly. The coordinating center and the trial steering committee will periodically check the evolution of the research, revising the data included by each center, and contact to them if needed. The main investigator will update the trial steering committee with any new information on the trial at an annual meeting (or ad hoc if requested).

The trial will be monitored and audited by the main investigators and sponsor.

The independent data monitoring committee will meet every 6 months or more often if required.

The ethics committee will evaluate the protocol only if modified by the main investigator and presented for evaluation. Any modification of the protocol must be approved by the ethics committee.

### Plans for communicating important protocol amendments to relevant parties (e.g., trial participants, ethical committees) {25}

In case of trial modification, it will need to be approved by the IEC of the coordinating center and modify in ClinicalTrials.gov. Main investigator of the coordinating center will inform about the changes to the rest of the hospitals involved. All changes will be modified in the protocol, adapting the version and date.

### Dissemination plans {31a}

The principal investigator declares his commitment to publish the final results of the study in a scientific publication, and the results will be showed in scientific conferences.

The ICMJE’s recommendations regarding authorship according to the following four criteria shall be taken into account:

1. Substantial contribution to the conception or design of the work or to the acquisition, analysis, or interpretation of data for the work
2. Drafting of the paper or critical revision of its important intellectual content
3. Final approval of the version to be published
4. Commitment to be responsible for all aspects of the work by ensuring that all questions concerning the accuracy or honesty of any part of the work have been properly investigated and resolved

All persons qualifying as authors according to the ICMJE will be asked to sign an authorship contract.

The inclusion of a minimum of 5 patients per center is required. In case of including 10 patients, the name of the collaborating author in that center will appear in the title of the future scientific publication (as long as they meet the criteria of ICMJE’s recommendation). The order of authors in the scientific publication will depend on the inclusion contribution of each one. If there happens to exist a limit on the number of authors in the scientific journal, the inclusion of the remaining authors will be assessed as “Breast Cancer Research Group.”

## Discussion

The project aims to investigate an alternative therapeutic strategy in BC patients undergoing AL, a highly prevalent disease around the world with a high care burden in our health system.

Assuming that seroma will always appear, inherent to the surgical procedure, we directed aims not to prevent its formation but to prevent its symptoms. With this aim in mind, we decided to investigate over the use of Glubran 2® in axillary surgery. Scientific literature reports good results when using Glubran 2® as a hemostatic agent (liver, kidney, spleen, pancreas) [13], as a sealant (blood vessels, lymphatic vessels, anastomosis), an adhesive (abdominal or inguinal meshs), as a shutter of fistula tracts [11], and in many other surgical procedures. So, we chose this product for its sealant, adhesive, and hemostatic properties, thinking that it would help us reduce the death space but also would control of lymphorrhea and seroma formation in the axillary cavity.

Our main objective is to determine if the use of this sealing product has any role in diminishing the seroma formation; another is to find out if Glubran 2® is capable of reducing the pain and other symptoms related to AL. All patients will keep a close follow-up, and periodical QoL test will be held, in order to detect any major discomfort as quickly as possible.

Performing an AL is a common and regulated procedure in all breast cancer centers; therefore, there should not be a difference in this practice between hospitals. Moreover, being this study multicenter and international improve its external validity.

Where there is a difference between hospitals, regions, and countries is in relation to the postoperative regime. In our hospital, patients undergoing an AL (who are ASA < 4 and absence of adequate cognitive capacity or family support) no longer stay in the hospital. Since 2021, we perform a protocol of outpatient surgery with the collaboration of “Day Hospital Unit,” so that patients can sleep at their homes the day of the surgery, the following day receive a visit of a doctor and a nurse at home to asses’ pain control and seroma formation, and in a week come to the hospital for a clinical assessment by the surgeon. However, they can phone contact with the breast cancer nurse anytime in case of doubt. However, many of the participating hospitals perform AL in an impatient regime. Being this item non-relevant for the objectives of the study and having no impact in its results, we did neither collect nor analyze this data.

We intend to assess whether patients subject to AL without placement of drains have a correct QoL, with the aim of changing a paradigm, justifying and generalizing the non-benefit of using an axillary drain. The results of the present trial will be highly relevant and will allow a large number of patients undergoing an AL to benefit from a less morbid, less painful, but equally effective seroma management.

Although the current tendency in the treatment of BC is to reduce the number of AL practiced to our patients, caring about reducing the discomfort associated with the procedure and reducing the rate of clinical axillary seroma will for sure be appreciated by our patients.

## Trial status

The current protocol version is number 3, 16 March of 2022. First, the patient was recruited in the coordinating center (Mataró hospital) in March 2022. To date, eight hospitals have already started recruiting patients. The study will finish when the last patient completes the 12-month follow-up clinical visit. We calculate that patient recruitment will end by September 2024.

## Data Availability

At the end of the study, the statistician will have access to the data in order to analyze the results. During the study, the main investigator of the coordinating center will have access to all data.

## Abbreviations

AL: Axillary lymphadenectomy
BC: Breast cancer
VAS: Visual analogue scale
CRF: Case report form
eCRF: Electronic case report form
SAP: Statistical analysis plan
iDMC: Independent data monitoring committee
IEC: Investigation ethics committee
ICMJEs: International Committee of Medical Journal Editors
AEC: Asociación Española de Cirujanos
BCRG: Breast Cancer Research Group
QoL: Quality of life
MOS: Major outpatient surgery
